# HIV-free survival among breastfed infants born to HIV-positive women in northern Uganda: a facility-based retrospective study

**DOI:** 10.11604/pamj.2020.37.297.22928

**Published:** 2020-12-02

**Authors:** Irene Aguti, Charles Kimbugwe, Patricia Apai, Siraji Munyaga, Richard Nyeko

**Affiliations:** 1 Faculty of Medicine, Gulu University, Gulu, Uganda,; 2 Department of Paediatrics and Child Health, Faculty of Health Sciences, Lira University, Lira, Uganda

**Keywords:** HIV-free survival, mortality, HIV-infection, infants, breastfeeding, option B+

## Abstract

**Introduction:**

the HIV-free survival rate is the gold-standard measure of the effectiveness of interventions towards prevention of mother-to-child transmission of HIV in any setting. However, data on HIV-free survival among the HIV-exposed infants followed up in most low-resource settings are lacking. We determined the HIV-free survival among breastfed infants in two tertiary facilities in a resource-poor setting in northern Uganda.

**Methods:**

we conducted a retrospective cohort study in May 2019 and retrospectively reviewed records of HIV-exposed infants registered in 2014 through 2016 at two tertiary facilities in northern Uganda. We analyzed data using SPSS v16 software package. The chi-square and Student t-tests were used to compare factors among infant groups. Multivariate logistic regression analysis was used to determine factors independently associated with HIV-free survival. P-value <0.05 was considered for statistical significance.

**Results:**

majority of the infants were males 55.6% (203/365) and 98.6% (360/365) received nevirapine prophylaxis. A total of 345 (94.5%) infants were exclusively breastfed, only 100/345 (29.0%) of whom were exclusively breastfed for at least 6 months, while the breastfeeding status of 44/345 (12.8 %) infants could not be ascertained. The overall HIV-free survival rate was 93.7% (342/365), while 2.7% (10/365) were HIV-infected and 3.6% (13/365) died. Infants´ age at enrolment in care (aOR 5.20, p=0.008) and treatment facility (aOR 3.76, p=0.027) were the independent determinants of HIV-free survival.

**Conclusion:**

the HIV-free survival rate among the breastfed infants in the study setting marginally falls short of the recommended standard, thus calling for more efforts to improve survival.

## Introduction

Even though the incidence of paediatric HIV is falling as a result of an increase in effective methods to prevent mother-to-child transmission of HIV, an estimated 180,000 (110,000-260,000) new paediatric HIV-1 infections occurred in 2017 globally, primarily through mother-to-child transmission, mainly in sub-Saharan Africa [1]. In Uganda, a recent survey estimates that 95,000 children aged 0-14 years were living with HIV in 2017 [2], about 85,500 (90%) of them having acquired the infection through mother-to-child transmission (MTCT). Besides, Uganda registered an estimated 7,600 new paediatric HIV infections in 2017, with 3,800 (4.0%) AIDS-related deaths [1].

Without interventions for the prevention of mother-to-child transmission of HIV (PMTCT), the rate of maternal-to-child transmission (MTCT) of HIV during pregnancy and delivery is estimated at 15-25%, and the additional risk through breastfeeding is estimated at 5-20% [3, 4]. With interventions for PMTCT, the transmission reduces to <5% in the breastfeeding population and to <2% in the non-breastfeeding population, making the prevention of mother-to-child transmission of HIV (PMTCT) a major public health approach for reducing the scourge [5]. In this regards, the World Health Organization (WHO) infant feeding guidelines in the context of HIV infection recommend exclusive breastfeeding (EBF) for six months followed by complementary feeding and continued breastfeeding up to at least one year of age, under the cover of antiretroviral treatment (ART) to either the mother or the infant [6]. The objective of this is to minimize the risk of mother-to-child transmission of HIV as well as increase the survival of the infants by preventing death from diarrhoea, pneumonia and malnutrition. The HIV-free survival rate (the proportion of HIV-exposed infants who are alive and HIV uninfected at 18-24 months of age) is a key outcome used to measure the effectiveness of any PMTCT programs [7].

Uganda rolled out the nationwide implementation of PMTCT option B+ approach in 2013, a policy whereby all HIV-infected pregnant and breastfeeding women were started on lifelong combination ART (lamivudine/tenofovir disoproxil fumarate/efavirenz [3TC/TDF/EFV] as the preferred regimen), regardless of their clinical stage and/or CD4 cell count, following the WHO guidance [8]. This was later expanded in a 2016 update of the national guidelines [9] following the release of new WHO guidelines in 2015 [10], with the recommendation for lifelong ART for all HIV-positive individuals, including pregnant and breastfeeding women, regardless of clinical stage and CD4 count.

Although HIV-free survival rate is the gold-standard measure of the effectiveness of interventions towards prevention of mother-to-child transmission of HIV in any setting, data on HIV-free survival among the HIV-exposed infants followed up in most low-resource settings are lacking. This study assessed the HIV-free survival rate and determinants among infants born to HIV positive mothers in northern Uganda in both an urban and peri-urban resource-poor upcountry settings in light of the current efforts to the elimination of mother-to-child transmission of HIV (eMTCT).

## Methods

**Study design**: we conducted a retrospective cohort study of infants born to mothers living with HIV and registered for comprehensive care in 2014 through 2016 at two tertiary health facilities in Gulu District, northern Uganda. Infants who had complete follow up data and final status were included in the study, while those lost to follow up with unknown final status were excluded.

**Study setting**: this study was conducted at two tertiary referral hospitals in northern Uganda - Gulu regional referral hospital, a public health facility (PHF) in an urban setting and St. Mary´s Hospital Lacor, a private not-for-profit (PNFP) facility in a peri-urban setting. These are the two largest hospitals in the sub-region, serving the population from the entire northern region and beyond. Gulu District is located approximately 340 kilometres north of Uganda's capital city, Kampala. The population of Gulu District was estimated at 275,613 inhabitants [11]. Both hospitals run antiretroviral therapy (ART) clinics which provide HIV services to the people of Gulu District and beyond. Northern Uganda is a region recovering from over two decades of insurgency with a high burden of HIV (7.2%) compared to the national prevalence (6.2%) [2]. Access to health services remains a challenge. High levels of poverty and illiteracy, especially among women, is exacerbated by a high prevalence of preventable diseases, including HIV/AIDS.

**Study procedure and data collection**: records in the early infant diagnosis (EID) registers for a cohort of 422 HIV-exposed infants enrolled in care in 2014 through 2016 in the two facilities were retrospectively reviewed in May 2019 and data were abstracted for 365 using a standard data abstraction form containing variables selected based on the study objectives. Infants (57) who were lost to follow up with unknown final HIV status were excluded. The data abstraction was carried out by the researchers and double-checked daily for completeness and accuracy. We defined HIV-free survival in line with the WHO definition as “an infant or young child born to a mother living with HIV who remains both HIV uninfected and also alive at 18 months of age” [12].

**Determination of infants´ HIV status**: the infants´ final HIV status was based on documented test results routinely performed using national procedures and algorithms for early infant diagnosis. Within the period for which data was collected, the Uganda Consolidated ART Guidelines recommended that all infants born to HIV positive mothers be initiated on nevirapine prophylaxis at birth according to their risk classifications [9]. Infants were then routinely followed up at 6, 10, and 14 weeks, then monthly until 6 months of age and every 3 months until 18 months of age. The HIV status of infants was determined using the national algorithms for EID, and tested by polymerase chain reaction (PCR) for the detection of viral deoxyribonucleic acid (DNA) using dried blood samples (DBS) collected on filter paper at 4-6 weeks of age or at the earliest time thereafter [9]. For infants with an initial negative DNA PCR test result, a second DNA PCR test was performed 6 weeks after cessation of breastfeeding. Infants with an initial HIV DNA PCR positive test result had a confirmatory PCR performed at the time of ART initiation. A rapid HIV antibody testing was conducted at 18 months of age for all infants who test negative at first or second PCR to determine the final HIV status. The national guidelines recommended that all children below the age of 15 years who were confirmed HIV infected are immediately initiated on ART regardless of WHO clinical stage or CD4 count/percentage [9, 13]. Infants with negative DNA PCR test results had an HIV rapid test performed at 18 months of age to determine the final status. The recommendation for maternal ART within the time for which data were collected was based on option B+ where pregnant and breastfeeding women were started on lifelong 3TC/TDF/EFV as the preferred regimen.

**Data management and analysis**: data were entered, cleaned and analysed using Statistical Package for Social Scientists software package (SPSS for Windows, Version 16.0. Chicago, SPSS Inc.). In univariate analysis, categorical variables were summarized as proportions, while continuous variables as medians (interquartile range) or means and standard deviations (SD). HIV-free survival rate was calculated as the proportion of study infants who were alive with final HIV-negative status at 18 months, the denominator being all infants considered in the study. In the bivariate analysis, the chi-square test (for categorical variables) and Student t-test (for continuous variables) were used to test if the factors among infants with negative HIV status at 18 months were different from those among infants who became HIV-infected or died. Multivariate logistic regression analysis was used to determine the factors independently predicting HIV-free survival. Odds ratios with their 95% confidence interval (CI) were used to measure the strength of association between the outcome and predictor variables. Covariates with a p-value less than 0.2 and those with scientific plausibility (even if the p-value was greater than 0.2) were included in the multivariate analysis, and included age at enrolment in care, gender, treatment facility, maternal CD4 count, and timing of maternal HIV diagnosis and ART. P-value <0.05 was considered for statistical significance.

**Ethical considerations**: the study was approved by the Gulu University Research and Ethics Committee [approval number GUREC-046-18] and permission to access records was granted by the administrations of the respective hospitals.

## Results

**Description of the study population**: data were analyzed for 365 (86.5%) of the 422 HIV-exposed infants enrolled in care in two ART facilities in 2014 through 2016. Fifty-seven infants were lost to follow up without documented final HIV status and were excluded. The median age of the infants at enrollment in care was 1.5 months (IQR 1.5>-2.0) and more than half, 55.1% (201/365) were from a public health facility. Majority of the infants, 55.6% (203/365) were males and 98.6% (360/365) received nevirapine prophylaxis, 98.9% (356/360) of whom were initiated on prophylaxis within the desired 72 hours of birth (Table 1). A total of 345 (94.5%) infants were exclusively breastfed, only 100/345 (29.0%) of whom were exclusively breastfed for at least 6 months as per standard recommendations, while the breastfeeding status of 44/365 (12.1 %) infants could not be ascertained due to lack of documentation. Overall, 84.9% (310/365) of the infants were breastfed for at least 12 months or more. The median duration of exclusive and total breastfeeding was 5.0 months (IQR 4.0-6.0) and 14.0 months (IQR 13.0-15.0) respectively. The basic maternal characteristics were as summarized in Table 1. The majority, 63.3% (231/365) of the mothers were known HIV positives before pregnancy and more than half 55.6% (203/365) of the women were on ART before pregnancy. Only 12.1% (44/365) of the mothers had documented viral load, while 65.8% (240/365) had documented CD4 count with a mean CD4 count of 592.94 ± 360.56 cells/ml.

**Table 1:** baseline characteristics of the study population

Characteristics	Number	Percentage (%)
**Infant characteristics**		
Median age in months (IQR)	1.5 (1.5-2.0)	
**Final outcome**		
Discharged negative	342	93.7
HIV infected	10	2.7
Died	13	3.6
**Gender**		
Female	162	44.4
Male	203	55.6
Timelines of enrolment		
Within 2 months	317	86.8
Beyond 2 months	48	13.2
**Type of treatment facility**		
Private not-for-profit	164	44.9
Public health facility	201	55.1
**Nevirapine prophylaxis**		
Yes	360	98.6
No	5	1.4
**Timing of nevirapine**		
Within 72 hours	356	98.9
After 72 hours	2	0.55
Unknown timing	2	0.55
**Duration of exclusive breastfeeding**		
≥ 6 months	100	27.4
˂ 6 months	221	60.5
Unknown	44	12.1
**Total duration of breastfeeding**		
≥ 12 months	310	84.9
˂ 12 months	15	4.1
Unknown	40	11.0
**Maternal baseline characteristics**		
Mean CD4 (SD)*	592.94 (360.56)	
CD4 Count:	133	36.5
>500cells/ml	107	29.3
<500cells/ml	125	34.2
**Viral Load (VL)**		
≤1000cps/ml	39	10.7
≥1000cps/m	5	1.4
**HIV diagnosis**		
Before pregnancy	231	63.3
In antenatal/postpartum	134	36.7
**ART initiation**		
Before pregnancy	203	55.6
In antenatal/postpartum	162	44.4

* SD=Standard deviation

**Figure 1:**
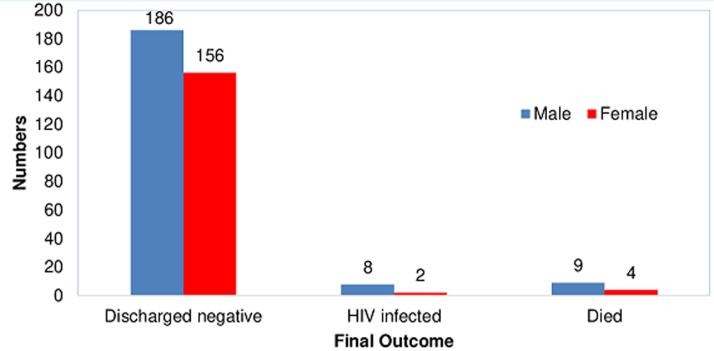
final outcome disaggregated by gender

**Figure 2:**
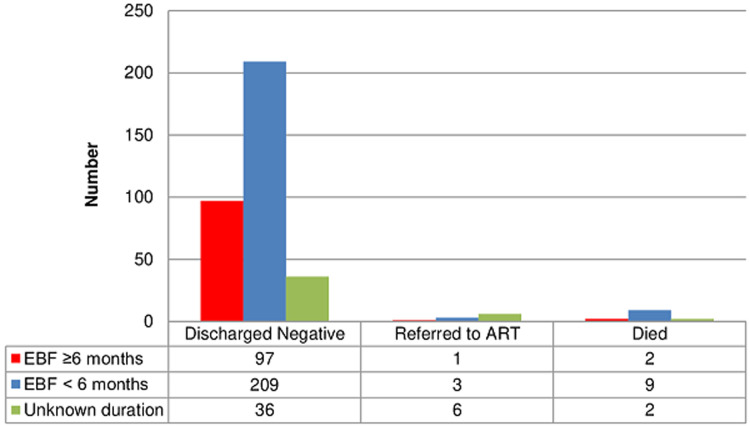
final outcome disaggregated by duration of exclusive breastfeeding

**HIV-free survival**: the 18-months HIV-free survival rate among HIV-exposed infants in the current study was 93.7% (342/365) and this was calculated only among infants whose final status was known. The overall HIV-infection and mortality rates were 2.7% (10/365) and 3.6% (13/365) respectively (Table 1). On further analysis of the infants who died or became infected, the mean age at death or HIV-infection for infants in the private not-for-profit (PNFP) facility was 5.6±7.5 months versus 2.3±2.5 months for those in the public health facility (PHF). Besides, a higher proportion of the infants who died or became HIV-infected were males 73.9% (17/23) compared to females 26.1% (6/23) (Figure 1). Likewise, only 13.0% (3/23) of the infants who died or became HIV-infected were exclusively breastfed for the recommended 6 months, while 52.2% (12/23) were exclusively breastfed for less than 6 months. The duration of exclusive breastfeeding for 8/23 (34.8%) infants who died or became infected could not be ascertained (Figure 2).

There was a statistically significant difference in the mean age of the infants who survived with a negative HIV status and those who were either HIV infected or died (F 6.06; p=0.014). Similarly, there was a statistically significant difference in HIV-free survival between infants who received care from the PNFP facility and those who received care from the public health facility (χ^2^5.34; p=0.021) (Table 2). There was a tendency to a better outcome among the female gender compared to males (96.3% vs. 91.6%) who in contrast were more likely to become infected or die (8.4% vs. 3.7%), although this difference did not reach a statistically significant level at bivariate analysis (χ^2^3.33; p=0.068). There was no statistically significant difference in HIV-free survival with regards to the rest of the infants´ characteristics and all of the maternal characteristics on the bivariate analysis (Table 2).

**Table 2:** infants and maternal characteristics associated with HIV-free survival

	HIV free N=342(%)	Infected/Died N=23(%)	χ^2^	P value
**Infant characteristics**				
Mean age (SD)	1.97 (1.66)	2.99 (4.11)	6.06ψ	0.014*
**Timelines of enrolment**				
Within 2 months	300 (94.6)	17 (5.4)	3.60	0.058
Beyond 2 months	42 (87.5)	6 (12.5)		
**Gender**				
Female	156 (96.3)	6 (3.7)	3.33	0.068
Male	186 (91.6)	17 (8.4)		
**Treatment facility**				
Private not-for-profit	159 (97.0)	5 (3.0)	5.34	0.021*
Public health facility	183 (91.0)	18 (9.0)		
**NVP prophylaxis**				
Yes	338 (93.9)	22 (6.1)	1.61	0.279
No	4 (80.0)	1 (20.0)		
**Timing of nevirapine				
Within 72 hours	335 (94.1)	21 (5.9)	0.13	1.000
After 72 hours	2 (100)	0 (0.0)		
**Duration of EBF^				
≥ 6 months	97 (97.0)	3 (3.0)	0.91	0.407
˂ 6 months	209 (94.6)	12 (5.4)		
**Total duration of BF^^				
≥ 12 months	304 (98.1)	6 (1.9)	0.30	1.000
˂ 12 months	15 (100)	0 (0.0)		
**Maternal characteristics**				
†CD4 Count				
>500cells/ml	126 (94.7)	7 (5.3)	0.94	0.331
<500cells/ml	98 (91.6)	9 (8.4)		
**†Viral Load**				
˂ 1000cps/ml	37 (94.9)	2 (5.1)	0.27	1.000
≥1000cps/m	5 (100)	0 (0.0)		
**HIV Diagnosis**				
Before pregnancy	218 (94.4)	13 (5.6)	0.48	0.487
During ANC/PNC	124 (92.5)	10 (7.5)		
**ART initiation**				
Before pregnancy	191 (94.1)	12 (5.9)	0.12	0.731
During ANC/PNC∂	151 (93.2)	11 (6.8)		

**χ^2^** = Chi-square (df=1); ψF statistic for comparing means; *P-value significant (<0.05); **Only those who received NVP and with a documented duration of EBF and the total duration of breastfeeding were considered. ^EBF=Exclusive breastfeeding; ˄˄Breastfeeding; †Only those with documented viral load and CD4 count. ∂ANC/PNC =Antenatal care/Postnatal care

On multivariate analysis, infants´ age at enrolment in care and the facility providing care and follow up remained statistically significant in determining HIV-free survival. Infants enrolled within 2 months of age were more likely to survive with a negative HIV status compared to those enrolled beyond 2 months of age (aOR 5.20, 95% CI 1.53-17.65; p=0.008). Likewise, infants enrolled and followed up in the private not-for-profit facility were more likely to have survived with a negative HIV status compared to those followed up in the public health facility (aOR 3.76, 95% CI 1.17-12.14; p=0.027) (Table 3). Females were twice as likely to have HIV-free survival as males but this was not statistically significant (aOR 2.18, 95% CI 0.70-6.75; p=0.179). There was a 72% increased likelihood of HIV-free survival among infants born to mothers with CD4 count ≥500cells/ml compared to infants born to mothers with CD4 count <500cells/ml but this was not statistically significant (aOR 1.72, 95% CI 0.58-5.06; p=0.327). Mothers with known HIV positive status before pregnancy were about thrice as likely to have infants discharged HIV negative as mothers who did not know their HIV status before pregnancy, although this was not statistically significant (aOR 2.97, 95% CI 0.35-25.06; p=0.318). There was an increased likelihood of HIV-free survival among infants whose mothers were on ART before conception in comparison to those whose mothers-initiated ART during pregnancy or in the postpartum period, but this also was not statistically significant (aOR 2.24, 95% CI 0.30-16.92; p=0.433) (Table 3).

**Table 3:** multivariate logistic regression for factors independently associated with HIV-free survival

Characteristics	AOR (95% CI)	P-value
Timeliness of enrolment		
Within 2 months	5.20 (1.53-17.65)	0.008*
Beyond 2 months	1.0	
**Gender**		
Female	2.18 (0.70-6.75)	0.179
Male	1.0	
**Treatment facility**		
Private not-for-profit	3.76 (1.17-12.14)	0.027*
Public health facility	1.0	
**CD4 count**		
≥500cells/ml	1.72 (0.58-5.06)	0.327
˂ 500cells/ml	1.0	
**Maternal HIV diagnosis**		
Before pregnancy	2.97 (0.35-25.06)	0.318
During Antenatal/Postnatal	1.0	
**Timing of maternal ART**		
Before pregnancy	2.24 (0.30-16.92)	0.433
During antenatal/Postnatal	1.0	

* P-value significant (<0.05), AOR=Adjusted Odds ratio, CI=95% confidence interval

## Discussion

**HIV-free survival**: in this study, we assessed the 18-months HIV-free survival among HIV- exposed infants from a largely breastfeeding population, followed up in two tertiary health facilities in a low-resource setting in northern Uganda under the PMTCT option B+ programme. The overall HIV-free survival rate in the current study among exposed infants was 93.7%, while 6.3% of the infants were either HIV-infected (2.7%) or died (3.6). The 18-months HIV-free survival rate of 93.7% found in this study would pass for being among the highest seen among breastfeeding population in the real-world African setting, and only marginally falls short of the >95% HIV-free survival rate recommended among breastfeeding populations [5]. Our finding mirrors that among exclusively breastfed infants in a rural Haiti of 93.7% [14] and compares well with the 93.2% 24-month HIV-free survival rate under similar PMTCT Option B+ programme reported by Gill and colleagues (2017) in Rwanda [15]. The rate in the current study is, however, lower than the 95.9% HIV-free survival rate reported in a community-based survey in Swaziland [7]. Importantly, our finding contrasts with and is higher than, the 89.8% and 85.8% pooled estimates for 12-month and 24-month HIV-free survival respectively as reported by Chikhungu *et al*. (2016) in a systematic review of 18 studies evaluating HIV-free survival among breastfed infants of HIV positive women on ART [16]. The rate in our study is also higher than the pre-Option A rates of 24-month HIV-free survival reported in South Africa (77.7%), Cameroon (72.6%), Zambia (83.1%) and Côte d´Ivoire (84.4%) [17]. The above differences could be explained by the differences in the guidelines or PMTCT approaches used in the different studies and the timing of measurements of HIV-free survival. Notwithstanding, these findings seem to lay credence to the effectiveness of option B+ over the earlier approaches towards the elimination of mother-to-child transmission of HIV among the breastfeeding population in developing countries.

The HIV transmission rate in our study shows a much lower rate compared to the 14% rate at 12 months earlier reported by Kagaayi and colleagues (2008) in Rakai, Uganda [18]. Our finding also shows a lower HIV-infection rate compared to the 15.7% and 11.8% HIV transmission rates at 6-weeks and 18-months respectively observed in the HIVNET 012 study in Uganda using single-dose maternal and infant nevirapine [19]. In a post-Option A study in Zimbabwe, the MTCT of HIV was estimated at 4.8% [20], while the cumulative risk for MTCT in a Malawian study at 24-months among breastfed infants who received a short regimen of ARV prophylaxis was 9.7% [21]. This difference could likely be due to the differences in the PMTCT programmes under which these studies are conducted - Option A in the earlier Ugandan and Zimbabwean studies where women only received ARV prophylaxis during pregnancy till one week after delivery, and Option B+ in the current study where women received lifelong ART. This highlights the impact of the efforts towards elimination of MTCT of HIV implemented initially as PMTCT Options A, B and B+, and now as “treat all”. The mortality rate of 3.6% in the current study was generally low and mirrors that in a community-based household survey in Swaziland [7], but higher than the 1.1% reported in Mma Bana trial in Botswana [22], a difference possibly explained by the fact that infants in the Mma Bana trial breastfed for a shorter duration of 6 months (median 5.8 months).

**Factors associated with HIV-free survival**: based on our findings, the infants´ age at enrolment in care and the treatment facility were the two significant determinants of HIV-free survival among the HIV exposed infants. Infants enrolled in care within 2 months of age were significantly 5.2 times more likely to survive with a negative HIV status than those enrolled in care beyond 2 months of age. Likewise, there was a significant 3.76-fold likelihood of HIV-free survival for infants enrolled in a private not-for-profit (PNFP), faith-based facility compared to those enrolled in a public health facility. We supposed this could relate to the fact that infants followed up in the PNFP facility were enrolled earlier into care (mean age 1.91 ± 1.55 months) - a factor positively associated with a better outcome, compared to those in the public health facility (mean age 2.14 ± 2.16 months). This is valid and is in accord with the WHO recommendation for early infant diagnosis and a finding by Berhan *et al*. (2014) in Ethiopia where infants with delayed DNA PCR tests had a 30% excess risk of mother-to-child transmission of HIV compared to those tested early [23]. However, this difference could also be as a result of several factors - both infant and caregiver factors as well as healthcare factors which could not be explored in this study.

There was a non-statistically significant influence of gender on the infants´ final outcome, with females being twice more likely to survive with a negative HIV status compared to their male counterparts, who were more likely to die or get HIV-infected. The difference in outcome among HIV exposed infants by gender has not been well explained in previous studies. However, one plausible view could relate to the fact that an increased risk of morbidity and mortality among young males, in general, has long been advanced, albeit with no well understood scientific explanations [24, 25]. In the context of HIV therefore, this could translate to faster disease progression in males compared to female infants.

The overall duration of exclusive breastfeeding and total breastfeeding did not significantly influence the HIV-free survival in the current study. However, infants who exclusively breastfed for at least 6 months had a higher rate of HIV-free survival compared to those exclusively breastfed for less than 6 months (97.0% vs. 94.6%), but this was not statistically significant (p=0.407). This finding compares well with that reported by Alvarez-Uria *et al*. (2012) in India [26] and Peltier *et al*. (2009) in Rwanda [27] where there was no significant difference in HIV-free survival with breastfeeding status. Besides, the Rwandan study estimated HIV-free survival at an earlier time interval of 9 months during a different approach to PMTCT and the infants were breastfed for only 5-6 months followed by rapid weaning. While the overall rate of exclusive breastfeeding in this study (94.5%) tends to compare with that reported by Okafor *et al*. (2014) in Nigeria of 91.8% [28], the fact that only very few mothers (29.0%) in the current study exclusively breastfed their infants adequately for at least 6 months could have far-reaching implications. This is evidenced in a report in a rural Ugandan study where breastfeeding for shorter than 6 months was associated with a 6-fold increased risk of mortality [29], consequent to deprivation from the protective benefit of exclusive breastfeeding against diarrhoea, malnutrition and other common childhood illnesses. This should, therefore, be of concern since it could be a precursor of general misinformation and inherent negative perception about breastfeeding in the context of HIV, which calls for more awareness and counselling.

More than one half (55.6%) of the women in our study were receiving ART before pregnancy, a finding consistent with a report from a previous study in Nigeria [30]. However, the timing of maternal HIV diagnosis and ART initiation did not significantly influence HIV-free survival in our study; although there was a lower HIV-infection or mortality rate among infants of mothers already on ART before pregnancy compared to that among infants of mothers initiated on ART during pregnancy or in the postnatal period (5.8% vs. 6.8%). This finding, although not statistically significant, is in keeping with that reported in a Nigerian study where the risk of HIV transmission was significantly lower among babies whose mothers commenced HAART before pregnancy (3.4%) compared to those whose mothers initiated HAART during pregnancy (5.4%) [31]. Similar benefits of prior maternal ART on PMTCT of HIV have been reported in Cameroon [32] and South Africa [33]. The above results could be explained by the fact that one of the hypothesized benefits of lifelong ART is the protection against HIV transmission in subsequent pregnancies, resulting from greater chances of virologic suppression. This was evidenced in a report by Gill *et al*. (2017) in a Rwandan study where a substantial proportion of women on ART before pregnancy had suppressed viral load (VL), a factor thought to have contributed to the high effectiveness of PMTCT [15]. Likewise, available shreds of evidence also point to the fact that long duration of ART may be associated with high viral load (VL) or viral rebound postpartum which could be associated with increased risk of mother-to-child transmission [15, 34, 35], supporting the importance of VL monitoring during pregnancy and breastfeeding, and continued adherence counselling [15]. In our study, data on maternal VL suppression was very limited (VL was not widely available in Uganda within the period for which data was collected for this study), making a comparison of HIV-free survival based on maternal VL impossible.

**Limitations of the study**: the main limitation of the current study arises from its use of retrospective data where missing data limited the depth of the study. Secondly, exclusion of those lost to follow up whose final HIV status could not be ascertained is likely to have created some selection bias, affecting the accuracy of the estimates of survival in the current study. Our estimates may, therefore, be an overestimate or underestimate, and our results could, therefore, be skewed since, for instance, it is likely that those infants lost with a missing final status more frequently died than remained HIV-free and survived.

## Conclusion

The HIV-free survival rate of 93.7% among the cohort of breastfed infants in the current study was relatively high but falls short of the acceptable rate of greater than 95% among breastfeeding population. This rate could also be an overestimate, however, because the infants lost to follow up with missing final status who were not considered in this study could be assumed to more frequently have died than remained HIV-free and survived. This calls for more concerted efforts especially in ensuring that infants are retained in care for as long as is required. A comprehensive prospective study to provide a more accurate measure of success would be recommended.

### What is known about this topic

- Exclusive breastfeeding for 6 months followed by complementary feeding until 12-24 months and maternal ART improves survival while reducing the risk of MTCT of HIV;
- Maternal viral load suppression is one of the most important determinants of MTCT of HIV.

### What this study adds

- This study highlights the change overtime in PMTCT interventions and outcome within the current recommendations relative to previous recommendations;
- It also adds the knowledge of HIV-free survival among exposed infants in resource-limited settings.
